# Epidemiology of Extrapulmonary Tuberculosis among Inpatients, China, 2008–2017

**DOI:** 10.3201/eid2503.180572

**Published:** 2019-03

**Authors:** Yu Pang, Jun An, Wei Shu, Fengmin Huo, Naihui Chu, Mengqiu Gao, Shibing Qin, Hairong Huang, Xiaoyou Chen, Shaofa Xu

**Affiliations:** Beijing Chest Hospital, Capital Medical University, Beijing, China

**Keywords:** tuberculosis, tuberculosis and other mycobacteria, bacteria, extrapulmonary, drug resistance, risk factor, China, inpatients, epidemiology

## Abstract

We investigated the epidemiology of extrapulmonary tuberculosis (TB) among patients admitted to Beijing Chest Hospital, Beijing, China, during January 2008–December 2017. Of 19,279 hospitalized TB patients, 33.4% (6,433) had extrapulmonary TB and 66.6% (12,846) had pulmonary TB. The most frequent forms of extrapulmonary TB observed were skeletal TB (41.1%) and pleural TB (26.0%). Younger, female patients from rural areas were more likely to have extrapulmonary TB. However, patients with diabetes mellitus were less likely to have extrapulmonary TB compared with patients without diabetes. A higher proportion of multidrug-resistant (MDR) TB was observed among patients with extrapulmonary TB than among patients with pulmonary TB. We observed a large increase in MDR TB, from 17.3% to 35.7%, for pleural TB cases. The increasing rate of drug resistance among extrapulmonary TB cases highlights the need for drug susceptibility testing and the formulation of more effective regimens for extrapulmonary TB treatment.

Tuberculosis (TB) is a major public health concern worldwide (*1*). The World Health Organization (WHO) estimated 10.4 million incident cases of TB and 1.67 million TB deaths in 2017 (*1*). Although TB most commonly affects the lungs, it also can affect other sites, a form known as extrapulmonary TB (*2*). The most common anatomic sites affected by extrapulmonary TB are lymph nodes, pleura, bone and joints, urogenital tract, and meninges (*3*). Several types of extrapulmonary TB, such as tuberculous meningitis and miliary TB, cause substantial rates of illness and death in various populations (*4*). Of the 6.3 million new TB cases recognized by WHO in 2017, 16% were extrapulmonary TB cases; incidence rates ranged from 8% in the Western Pacific Region to 24% in the Eastern Mediterranean Region (*1*). In the past few decades, studies from high-income countries have shown that extrapulmonary TB cases comprise an increased proportion of total TB cases (*5*,*6*). Despite this data, research on extrapulmonary TB is limited, possibly because extrapulmonary TB is less transmissible than pulmonary TB (*6*–*8*).

Because extrapulmonary TB can affect virtually any organ, it produces a wide spectrum of clinical manifestations that pose challenges to effective disease diagnosis and management (*3*,*9*). In general, extrapulmonary TB affects persons with diabetes and HIV, as well as young children (<15 years of age) and older adults (>65 years of age) (*10*). Recent studies have revealed that women and persons who migrate from areas of high TB incidence are at greater risk for extrapulmonary TB (*10*–*12*). In addition, extrapulmonary TB anatomic sites exhibit variability related to patient geographic location, population group, and a wide variety of host factors (*13*,*14*). Previous studies conducted on extrapulmonary TB have been in high-income countries, thus indicating the need to understand extrapulmonary TB in low- and middle-income countries.

China has the second highest number of TB cases in the world, accounting for ≈9% of global TB incidence (*1*). Nationwide survey data indicated that China has reduced smear-positive TB prevalence by >50%, from 170 cases/100,000 population in 1990 to 59 cases/100,000 population in 2010 (*15*). Despite past success in controlling pulmonary TB, limited available epidemiologic information indicates that extrapulmonary TB incidence may be increasing in China (*16*). In this study, we retrospectively reviewed the clinical manifestations of extrapulmonary TB in hospitalized patients at Beijing Chest Hospital (Beijing, China) during January 2008–December 2017. Our aim was to analyze the proportions of various extrapulmonary TB forms and to identify independent risk factors associated with the occurrence of extrapulmonary TB.

## Materials and Methods

### Data Sources and Collection

We performed a descriptive analysis of demographic and clinical data of inpatients treated for extrapulmonary TB at Beijing Chest Hospital from January 1, 2008, through December 31, 2017. Beijing Chest Hospital, designated a National Clinical Center on Tuberculosis, is a 900-bed hospital that delivers specialized treatment for TB and thoracic tumors. This hospital provides tertiary care for TB patients from Beijing municipality and parts of surrounding regions and accepts severe TB case referrals for patients originating from other regions of China. In addition, the hospital, under the oversight of the hospital authority, ensures compliance with strict protocols for patient care. 

Patients who come to the hospital with TB symptoms and who are presumed to have TB disease are asked to provide 2 sputum samples for laboratory testing. Samples are routinely tested for pulmonary and extrapulmonary TB, including microscopic examination, mycobacterial culture, or molecular testing. In addition, tissue specimens also are analyzed in the histology laboratory. Active TB disease is diagnosed by meeting >1 of the following criteria: 1 smear-positive or culture-positive specimen; positive histology (i.e., the presence of acid-fast bacilli in Zeihl-Neelsen–stained histological section); or TB history and strong clinical and radiographic evidence supporting the presence of active TB. The diagnosis of extrapulmonary TB followed the definitions and categories defined in the national guidelines for diagnosis of extrapulmonary TB in China (*17*). 

During the timeframe of our analysis, 20,534 patients meeting the criteria for active TB were hospitalized at Beijing Chest Hospital. Hospitalization criteria included TB requiring surgery, severe or complicated TB, and admission at patients’ request. Cases are categorized by major disease anatomic site and reported as either pulmonary TB or extrapulmonary TB. For our study, we excluded patients who were co-infected with pulmonary TB and extrapulmonary TB (1,255/20,534) from our descriptive statistics and risk factor analyses because their illnesses were not distinctly classifiable as either pulmonary TB or extrapulmonary TB (*5*). According to classification schemes described in previous reports, the extrapulmonary TB group included any extrapulmonary disease forms (i.e., pleural, lymphatic, skeletal, genitourinary, meningeal, and others) (*5*). In our study, disseminated TB refers to active TB detected in >1 noncontiguous anatomic site, miliary TB, or *Mycobacterium tuberculosis* isolated from blood samples. 

The hospital’s electronic patient record system documented inpatient illness, treatment, and care over time. We collected multiple demographic and clinical variables from electronic patient records to conduct comparative analyses between extrapulmonary TB and pulmonary TB groups, including sex, age, ethnicity, place of residence, previous TB episode, and concurrent conditions. Beijing Chest Hospital screens all patients for diabetes, according to national guidelines. These guidelines stipulate a fasting blood glucose test be performed by using venous plasma and a biochemical analyzer; a fasting blood glucose value ≥7.0 mm (126 mg/dL) was deemed a positive result for diabetes (*18*). 

The hospital’s clinical laboratory tested all positive mycobacterial cultures for drug susceptibility by using an absolute concentration method, according to WHO guidelines (*19*). Because clinical laboratories have performed routine drug-susceptibility testing for first- and second-line drugs since the 1990s, we compared extrapulmonary TB and pulmonary TB cases on the basis of previously established in vitro drug-susceptibility profiles for multidrug-resistant TB (MDR TB, defined as resistant to both rifampin and isoniazid) and extensively drug-resistant TB (XDR TB, defined as MDR with additional resistance to any fluoroquinolone and second-line injectable drug). 

### Statistical Analysis

We tabulated numbers and proportions of cases along with various demographic and clinical factors contributing to TB incidence and used univariable and multivariable logistic regression models to investigate factors associated with extrapulmonary TB. Multivariable models were built by using forward stepwise logistic regression procedures (with inclusion if p<0.05). For each anatomic site of disease, we tabulated the proportion of TB cases also by the year that a patient sought treatment. To analyze trends in the proportion of cases by anatomic site of disease, we used the χ^2^ trend test. We performed all calculations by using SPSS version 17.0 for Microsoft Windows (SPSS Inc., http://www.spss.com.hk). For p values <0.05, differences in distribution of categorical variables across various classifications were evaluated for statistical significance. A p value <0.05 for the χ^2^ trend test indicated that there was a significant change in the proportions of pulmonary TB and extrapulmonary TB over the duration of the study period. 

### Ethics Statement

This study was approved by the ethics committee of Beijing Chest Hospital (grant no. 2016-29), which is affiliated with Capital Medical University. This study used data collected from patient records while maintaining patient anonymity. Because this study presented no more than minimal risk of harm to patient subjects, the institutional review board approved a waiver of patient informed consent.

## Results

### Extrapulmonary TB Cases

**Figure 1:**
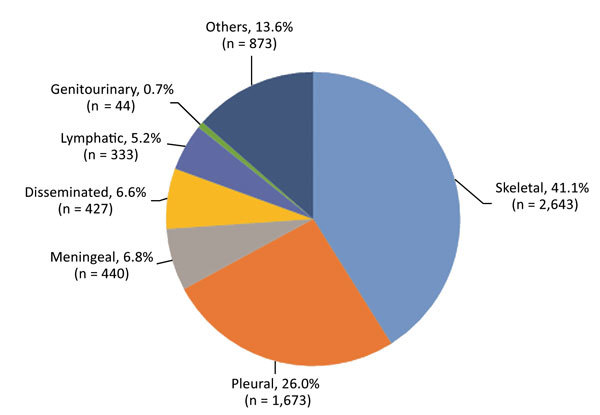
Extrapulmonary tuberculosis disease sites among 6,433 patients in China, 2008–2017.

During 2008–2017, a total of 20,534 patients with TB were hospitalized in Beijing Chest Hospital (Table 1). Of these patients, 62.6% (12,846) had pulmonary TB, 31.3% (6,433) had extrapulmonary TB, and 6.1% (1,255) had concurrent extrapulmonary TB and pulmonary TB. We excluded patients with concurrent extrapulmonary TB and pulmonary TB from our analyses because the primary site for TB infection could not be determined. Of the 6,433 extrapulmonary TB cases included in our study, the most frequent forms were skeletal TB (41.1% [2,643]) and pleural TB (26.0% [1,673]). Additional forms of extrapulmonary TB were meningeal TB (6.8% [440]), disseminated TB (6.6% [427]), and lymphatic TB (5.2% [333]) (Figure 1).

**Table 1:** General characteristics of pulmonary TB and extrapulmonary TB patients, China, 2008–2017*

Characteristic	No. (%) patients			Crude OR (95% CI)	p value	Adjusted OR (95% CI)	p value
	Pulmonary TB	Extrapulmonary TB	Total				
Sex							
M	8,757 (70.1)	3,736 (29.9)	12,493	Referent	–	Referent	–
F	4,089 (60.3)	2,697 (39.7)	6,786	1.55 (1.45–1.65)	<0.01	1.37 (1.27–1.47)	<0.001
Age group, y							
<25	2,024 (56.4)	1,563 (43.6)	3,587	2.51 (2.28–2.77)	<0.01	1.72 (1.53–1.93)	<0.001
25–44	3,539 (62.7)	2,102 (37.3)	5,641	1.93 (1.77–2.11)	<0.01	1.55 (1.40–1.73)	<0.001
45–64	4,078 (69.6)	1,782 (30.4)	5,860	1.42 (1.30–1.56)	<0.01	1.30 (1.17–1.44)	<0.001
≥65	3,205 (76.5)	9,86 (23.5)	4,191	Referent	–	Referent	–
Residence							
Urban	9,789 (68.3)	4,537 (31.7)	1,4326	Referent	–	Referent	–
Rural	3,057 (61.7)	1,896 (38.3)	4,953	1.34 (1.25–1.43)	<0.01	1.32 (1.22–1.43)	<0.001
Treatment history							
New case	10,967 (64.2)	6,118 (35.8)	17,085	Referent	–	Referent	–
Retreated case	1,879 (85.6)	315 (14.4)	2,194	0.30 (0.27–0.34)	<0.01	0.24 (0.20–0.27)	<0.001
Diabetes							
No	12,502 (66.3)	6,352 (33.7)	18,854	Referent	–	Referent	–
Yes	344 (80.9)	81 (19.1)	425	0.46 (0.36–0.59)	<0.01	0.54 (0.41–0.70)	<0.001
Culture determination							
Negative	6,256 (54.8)	5,152 (45.2)	11,408	Referent	–	Referent	–
Positive	6,080 (88.9)	758 (11.1)	6,838	0.15 (0.14–0.17)	<0.01	0.16 (0.15–0.18)	<0.001
Smear determination							
Negative	8,343 (60.0)	5,570 (40.0)	13,913	Referent	–		
Positive	4,000 (95.8)	174 (4.2)	4,174	0.07 (0.06–0.08)	<0.01		
DST							
MDR	684 (89.2)	83 (10.8)	767	1.25 (0.97–1.61)	0.084		
XDR	322 (89.2)	39 (10.8)	361	1.25 (0.88–1.77)	0.216		
Other	3,716 (91.1)	361 (8.9)	4,077	Referent	–		

*DST, drug susceptibility testing; MDR, multidrug-resistant; OR, odds ratio; TB, tuberculosis; XDR, extensively drug-resistant.

### Demographic and Risk Factor Characteristics of Extrapulmonary TB Cases

We summarized characteristics of extrapulmonary TB patients compared with pulmonary TB patients (Table 1). More women were afflicted with extrapulmonary TB (39.7% [2,697/6,786]; adjusted odds ratio [aOR] 1.37, 95% CI 1.27–1.47]) than with pulmonary TB (29.9% [4,089/6,786]; aOR 1.37, 95% CI 1.27–1.47]). The distribution of extrapulmonary TB also differed among age groups. Using patients >65 years of age as a control group, we found that young persons (<25 years of age) were more likely to have extrapulmonary TB (aOR 1.72, 95% CI 1.53–1.93) and that patients exhibited decreasing extrapulmonary TB risk with increasing age (aOR 1.55, 95% CI 1.40–1.73 for patients 25–44 years of age; aOR 1.30, 95% CI 1.17–1.44 for patients 45–64 years of age). In addition, patients from rural areas had significantly higher odds of having extrapulmonary TB compared with those from urban areas (aOR 1.32, 95% CI 1.22–1.43). Extrapulmonary TB patients also were less likely to have a previous TB episode than did pulmonary TB patients (aOR 0.24, 95% CI 0.20–0.27). Patients with diabetes had lower risk for extrapulmonary TB than did patients without diabetes (aOR 0.54, 95% CI 0.41–0.70).

TB diagnosis was confirmed by a positive culture in 12.8% (758/5,910) of extrapulmonary TB cases, which was significantly lower than the percentage for pulmonary TB cases (49.3%, 6,080/12,336; p<0.01). Low rates of positive cultures were noted for several extrapulmonary TB forms, especially lymphatic TB cases (2.1%, 6/292) and meningeal TB cases (4.5%, 17/379). The highest rates of positive cultures were observed for pleural TB cases (12.5%, 198/1,581) and disseminated TB cases (23.9%, 88/368). Of 758 culture-positive extrapulmonary TB cases, 483 (63.7%) cultures yielded drug susceptibility results. Of note, we identified MDR TB in 17.2% (83/483) and XDR TB in 8.1% (39/483) of extrapulmonary TB cases. These rates were both significantly higher than the corresponding rates for pulmonary TB cases (14.3% for MDR TB, 6.8% for XDR TB; p<0.01).

We further summarized associations of extrapulmonary TB with various demographic and clinical characteristics (Tables 2, 3). Of the 6,433 extrapulmonary TB cases included in our study, women were more likely than men to have skeletal TB (aOR 1.64, 95% CI 1.49–1.81), disseminated TB (aOR 1.68, 95% CI 1.36–2.08), lymphatic TB (aOR 4.11, 95% CI 3.17–5.34), and genitourinary TB (aOR 5.74, 95% CI 2.77–11.90) but were less likely to have pleural TB (aOR 0.76, 95% CI 0.67–0.86). Patients from urban areas had a higher frequency of lymphatic TB (aOR 1.92, 95% CI 1.35–2.73) than patients from rural areas. By contrast, patients from rural areas were more likely to have skeletal TB (aOR 0.63, 95% CI 0.56–0.70), meningeal TB (aOR 0.68, 95% CI 0.54–0.86), and disseminated TB (aOR 0.69, 95% CI 0.54–0.87) than patients from urban areas. Of note, concurrent diabetes decreased pleural TB risk (aOR 0.29, 95% CI 0.16–0.54) but not risks for contracting other types of extrapulmonary TB.

**Table 2:** Distribution of extrapulmonary tuberculosis by demographic and clinical characteristics, China, 2008–2017

Characteristics	Skeletal, %	Pleural, %	Meningeal, %	Disseminated, %	Lymphatic, %	Genitourinary, %	Others, %
Sex							
M	1,446 (38.7)	1,178 (31.5)	263 (7.0)	219 (5.9)	101 (2.7)	10 (0.3)	519 (13.9)
F	1,197 (44.4)	495 (18.4)	177 (6.6)	208 (7.7)	232 (8.6)	34 (1.3)	354 (13.1)
Age groups, y							
<25	520 (33.3)	470 (30.1)	128 (8.2)	154 (9.9)	84 (5.4)	6 (0.4)	201 (12.9)
25–44	736 (35.0)	561 (26.7)	170 (8.1)	139 (6.6)	162 (7.7)	17 (0.8)	317 (15.1)
45–64	914 (51.3)	362 (20.3)	102 (5.7)	72 (4.0)	62 (3.5)	18 (1.0)	252 (14.1)
≥65	473 (48.0)	280 (28.4)	40 (4.1)	62 (6.3)	25 (2.5)	3 (0.3)	103 (10.4)
Residence							
Rural	941 (49.6)	385 (20.3)	128 (6.8)	130 (6.9)	47 (2.5)	13 (0.7)	252 (13.3)
Urban	1,702 (37.5)	1,288 (28.4)	312 (6.9)	297 (6.5)	286 (6.3)	31 (0.7)	621 (13.7)
Treatment history							
New case	2,585 (42.2)	1,631 (26.7)	417 (6.8)	406 (6.6)	304 (5.0)	42 (0.7)	733 (12.0)
Retreated case	58 (18.4)	42 (13.3)	23 (7.3)	21 (6.7)	29 (9.2)	2 (0.6)	140 (44.4)
Diabetes							
No	2,591 (40.8)	1,660 (26.1)	437 (6.9)	423 (6.7)	332 (5.2)	44 (0.7)	865 (13.6)
Yes	52 (64.2)	13 (16.0)	3 (3.7)	4 (4.9)	1 (1.2)	0	8 (9.9)
Culture determination							
Negative	2,204 (42.8)	1,383 (26.8)	362 (7.0)	280 (5.4)	286 (5.6)	35 (0.7)	602 (11.7)
Positive	277 (36.5)	198 (26.1)	17 (2.2)	88 (11.6)	6 (0.8)	4 (0.5)	168 (22.2)

**Table 3:** Multivariate analysis of risk factors for 7 types of extrapulmonary TB compared with pulmonary TB, China, 2008–2017*

Patient characteristic	Odds ratio (95% CI)						
	Skeletal, n = 2,643	Pleural, n = 1,673	Meningeal, n = 440	Disseminated, n = 427	Lymphatic, n = 333	Genitourinary, n = 44	Others, n = 873
Sex							
M	Referent	Referent	Referent	Referent	Referent	Referent	Referent
F	**1.64** **(1.49–1.81)**	**0.76** **(0.67–0.86)**	1.15 (0.93–1.43)	**1.68** **(1.36–2.08)**	**4.11** **(3.17–5.34)**	**5.74** **(2.77–11.90)**	**1.27** **(1.09–1.48)**
Age group, y							
<25	1.14 (0.97–1.33)	**2.26** **(1.88–2.70)**	**3.24** **(2.17–4.86)**	**2.32** **(1.65–3.26)**	**3.54** **(2.14–5.85)**	2.26 (0.46–11.70)	**1.92** **(1.45–2.54)**
25–44	1.08 (0.93–1.25)	**1.86** **(1.57–2.20)**	**3.06** **(2.09–4.49)**	1.38 (0.98–1.93)	**4.93** **(3.09–7.87)**	3.48 (0.76–15.93)	**2.06** **(1.59–2.66)**
45–64	**1.33** **(1.16–1.52)**	1.01 (0.85–1.21)	**1.65** **(1.11–2.46)**	0.78 (0.54–1.12)	**2.09** **(1.26–3.46)**	**5.85** **(1.33–25.78)**	**1.83** **(1.42–2.36)**
≥65	Referent	Referent	Referent	Referent	Referent	Referent	Referent
Residence							
Rural	Referent	Referent	Referent	Referent	Referent	Referent	Referent
Urban	**0.63** **(0.56–0.70)**	0.94 (0.82–1.07)	**0.68** **(0.54–0.86)**	**0.69** **(0.54–0.87)**	**1.92** **(1.35–2.73)**	0.91 (0.45–1.85)	**0.83** **(0.70–0.98)**
Treatment history							
New case	Referent	Referent	Referent	Referent	Referent	Referent	Referent
Retreated case	**0.06** **(0.04–0.09)**	**0.14** **(0.10–0.20)**	**0.24** **(0.14–0.40)**	**0.31** **(0.19–0.49)**	**0.36** **(0.22–0.58)**	0.29 (0.07–1.20)	0.95 (0.77–1.17)
Diabetes							
No	Referent	Referent	Referent	Referent	Referent	Referent	Referent
Yes	0.84 (0.61–1.16)	**0.29** **(0.16–0.54)**	0.25 (0.06–1.03)	0.62 (0.23–1.68)	0.18 (0.02–1.30)	–	**0.34** **(0.15–0.77)**
Culture determination							
Negative	Referent	Referent	Referent	Referent	Referent	Referent	Referent
Positive	**0.13** **(0.12–0.15)**	**0.15** **(0.13–0.18)**	**0.05** **(0.03–0.08)**	**0.36** **(0.29–0.47)**	**0.02** **(0.01–0.06)**	**0.14** **(0.05–0.38)**	**0.31** **(0.26–0.37)**
*The pulmonary TB group was set as a reference for each comparison in multivariate analysis. Odds ratios were adjusted for all variables used in this model. Bold indicates significance. TB, tuberculosis.							

### Trends of Different TB Forms

**Figure 2:**
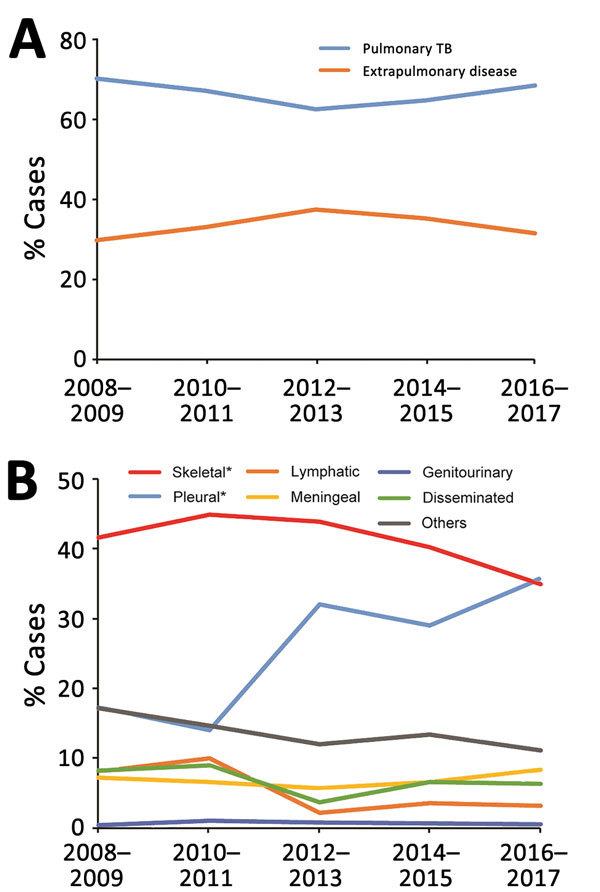
Trends in extrapulmonary TB and pulmonary TB, China, 2008–2017. A) Relative rates of extrapulmonary TB and pulmonary TB. B) Relative rates of different extrapulmonary TB forms. *p<0.01 compared with pulmonary TB group. Cases reported in 2-year periods. TB, tuberculosis.

We further analyzed trends by anatomic TB infection site from 2008 through 2017 (Figure 2). During the past decade, the proportion of extrapulmonary TB cases significantly increased from 29.8% to 31.4% (p<0.01) (Figure 2, panel A). Among extrapulmonary TB cases, the largest increase was seen in pleural TB, where the proportion of total extrapulmonary TB cases increased from 17.3% to 35.7% (p<0.01). A significant decrease in percentage of lymphatic TB within the total pulmonary TB case burden was observed (8.1% in 2008 vs. 3.2% in 2017; p<0.01). Meanwhile, we found no considerable differences in relative proportions of other anatomic sites in extrapulmonary TB disease between 2008 and 2017 (Figure 2, panel B).

## Discussion

We describe the epidemiologic and clinical characteristics of extrapulmonary TB patients in a hospital in northern China. Our data show that skeletal TB is the predominant form of extrapulmonary TB among inpatients in this region, accounting for ≈41% of all extrapulmonary TB cases. Previous reports have indicated significant differences in relative frequencies of anatomic sites of infection by geographic region. For instance, lymph nodes are the most common site of extrapulmonary TB in the Netherlands (39%), the United States (40%), and the United Kingdom (37%) (*2*,*5*,*9*), whereas pleural TB is the most prevalent form of extrapulmonary TB in Poland (36%) and Romania (58%) (*3*). We suggest two possible reasons for the disparity in the predominant site of extrapulmonary TB in China compared with reports from other countries. First, bacillus Calmette-Guérin (BCG) immunization provides differential protective efficacy against various forms of TB but is not widely administered in many countries (*20*,*21*). Therefore, we hypothesize that nationwide BCG immunization in China may be associated with different affected sites of extrapulmonary TB compared with nations in which BCG is not administered. Second, surveillance studies conducted in the European Union and Benin have shown that lymphatic TB is more frequently observed in children <15 years of age (*6*,*8*). Such age-related differences in lymphatic TB may partially be explained by factors involved in cellular immune system development and maturation (*22*). However, the lower frequency of lymphatic TB observed in this study may be because of the small sample size of younger patients compared with those of other age groups.

Consistent with other studies, our study found an association between extrapulmonary TB and either female sex or absence of previous TB episode (*2*,*5*). We also found the prevalence of extrapulmonary TB substantially decreases with advancing age, whereas the reverse is true for pulmonary TB. These opposing trends may be related to the dynamic changes in immunity during aging (*23*). Previous investigations have demonstrated functional decline of monocytes and macrophages during aging, whereas the production of proinflammatory cytokines by mononuclear cells in older persons (mean age 80.8 ±2.1 years) is higher than in young persons (mean age 26.8 ±0.8 years) (*24*). Nevertheless, our findings indicate that the mechanism by which *M. tuberculosis* affects extrapulmonary sites might involve different processes in extrapulmonary TB compared with those in pulmonary TB. Further research is needed to investigate the role of various immune responses to *M. tuberculosis* infection within different anatomic sites.

In addition, we found an association between diabetes and increased pulmonary TB incidence but not increased extrapulmonary TB incidence. Our findings are consistent with the results of studies from Brazil and Taiwan (*7*,*25*), but opposing results have been reported in the United States, where diabetes appears to increase extrapulmonary TB risk (*26*). Previous studies have shown that extrapulmonary TB may be associated with immunosuppression more than is pulmonary TB (*27*,*28*). However, concurrence of diabetes and TB might result in abnormalities of innate immune function, thus leading to increased extrapulmonary TB incidence within this population group, as seen in studies of patients with concurrent HIV and TB infections (*10*). An additional recent report from the southeastern United States revealed that immunosuppression was strongly and positively associated with risk for TB infection at meningeal and disseminated sites and less likely associated with risk for pleural TB (*29*). In view of these observations, the contradictory conclusions regarding the association between extrapulmonary TB and diabetes in previous studies may be related to geographic factors that influence the relative proportions of various extrapulmonary TB forms in patients living in different regions.

The increasing trend in pleural TB observed during the past decade should also be discussed here. In a previous study conducted in San Francisco, California, USA, Ong et al. found that pleural TB differs from other forms of extrapulmonary TB and exhibits the highest clustering rate of all forms of TB, suggesting that pleural TB clustering could be an indicator of recent transmission (*30*). The increasing epidemic of pleural TB recently observed in China may be driven by primary transmission. In view of this point, our data emphasize the importance of studying pleural TB and understanding the implications of this disease for achieving TB control in China.

In contrast to studies from countries with low TB incidence (*5*,*6*), we observed a higher proportion of MDR TB in extrapulmonary TB cases than in pulmonary TB cases in China. Numerous epidemiologic studies have documented that Beijing genotype strains, especially MDR TB, are strongly associated with drug resistance, suggesting increased bacterial fitness (*31*). We speculate that the higher frequency of MDR TB among extrapulmonary TB cases might be attributed to the current epidemic of the Beijing genotype in China. Despite a lack of experimental evidence, several studies regarding the molecular characteristics of extrapulmonary TB strains in China demonstrated the presence of the Beijing genotype in 88% of skeletal TB cases and in 80.0% of meningitis TB cases, rates that are higher than the average rate (62%) of the Beijing genotype among pulmonary TB cases in China (*32*,*33*). Because of the challenges of diagnosing and obtaining positive cultures for extrapulmonary TB, treating patients for this disease has been mainly empirical rather than based on drug susceptibility patterns of infecting strains. Such empirical treatment for patients with extrapulmonary TB no doubt delays effective treatment and may too often lead to a poor prognosis. Recently, Xpert MTB/RIF (Cepheid, http://www.cepheid.com), an automatic molecular assay, has been endorsed by the WHO based on a systematic review demonstrating its excellent performance for detecting *M. tuberculosis* and rifampin resistance in various types of specimens (*34*). Further investigation validates its use for diagnosis of extrapulmonary TB (*35*). In view of the high prevalence of drug resistance in extrapulmonary TB, our data suggest that the application of Xpert MTB/RIF is essential for the formulation of appropriate treatment regimens for patients with extrapulmonary TB in China.

Our study is subject to some limitations. First, our retrospective research only collected data of extrapulmonary TB cases from Beijing Chest Hospital rather than from national surveillance data, possibly limiting the overall relevant scope of our findings. Unfortunately, because extrapulmonary TB does not contribute substantially to the transmission of TB, it has been neglected by China’s National TB Control Program, and consequently the acquisition of national surveillance data has been difficult. Further surveys on the basis of data from TB hospitals located in different regions will be essential for obtaining improved extrapulmonary TB estimates in China. Second, only 12.8% (758/5,910) of patients with extrapulmonary TB yielded positive bacterial cultures, compared with 49.3% (6,080/12,336) of patients with pulmonary TB. Thus, most extrapulmonary TB was diagnosed only from clinical symptoms. Extrapulmonary TB can manifest with a variety of clinical symptoms that can mimic symptoms of other pathogens. Therefore, the lack of laboratory verification of extrapulmonary TB cases might lead to diagnostic delays, misdiagnoses, resistant strains, and increased mortality rates (*3*). Our data highlight the urgent need for a more accurate test, such as Xpert MTB/RIF or loop‐mediated isothermal amplification, to diagnose various forms of extrapulmonary TB. Third, use of new molecular diagnostics, such as Xpert MTB/RIF, likely would have improved extrapulmonary TB patient detection and possibly affected the analysis results. Although Xpert MTB/RIF is endorsed for testing specific specimens from patients suspected of having extrapulmonary TB, the diagnostic criteria of extrapulmonary TB remained unchanged during the study period because Beijing Chest Hospital was unable to obtain a license from the Chinese Food and Drug Administration for its use in smear-negative specimens. Fourth, our study included only inpatients in the final analysis. Rates of extrapulmonary TB obtained for this report might be affected by the higher rates of hospitalization for some types of extrapulmonary TB, such as skeletal and meningeal TB, due to severe clinical symptoms. Fifth, HIV infection has been considered an additional risk factor for extrapulmonary TB. However, patients co-infected with TB and HIV were referred to other hospitals that specialize in HIV treatment. The absence of patients co-infected with HIV and TB in this study precluded analysis of associations between extrapulmonary TB and HIV infection trends in China.

In conclusion, this study describes the epidemiologic and clinical characteristics of patients with extrapulmonary TB in a hospital in China. Our data show that the most frequent forms of extrapulmonary TB are skeletal TB and pleural TB. Women, young persons (<25 years of age), and persons living in rural regions are at high risk for developing extrapulmonary TB, whereas patients with diabetes have lower extrapulmonary TB risk compared with patients without diabetes. We also observed that most extrapulmonary TB is diagnosed from clinical symptoms, suggesting a high likelihood of diagnostic delays and misdiagnosis of extrapulmonary TB cases. In addition, the increased trend of drug-resistant TB among extrapulmonary TB highlights the importance of drug susceptibility testing in successful development of effective treatment regimens for patient with extrapulmonary TB in China.
